# Supplementary material for: Multimodal profiling reveals tissue-directed signatures of human immune cells altered with age
Source: Nat Immunol. 2025 Aug 13;26(9):1612–25. doi: 10.1038/s41590-025-02241-4 (PMC12396968; doi:10.1038/s41590-025-02241-4)
Supplement: Supplementary file 1 — Supplementary Figs. 1–10. [file 41590_2025_2241_MOESM1_ESM.pdf]

nature immunology

Supplementary information
https://doi.org/10.1038/s41590-025-02241-4

Multimodal profiling reveals tissue-directed signatures of human immune cells altered with age

In the format provided by the authors and unedited

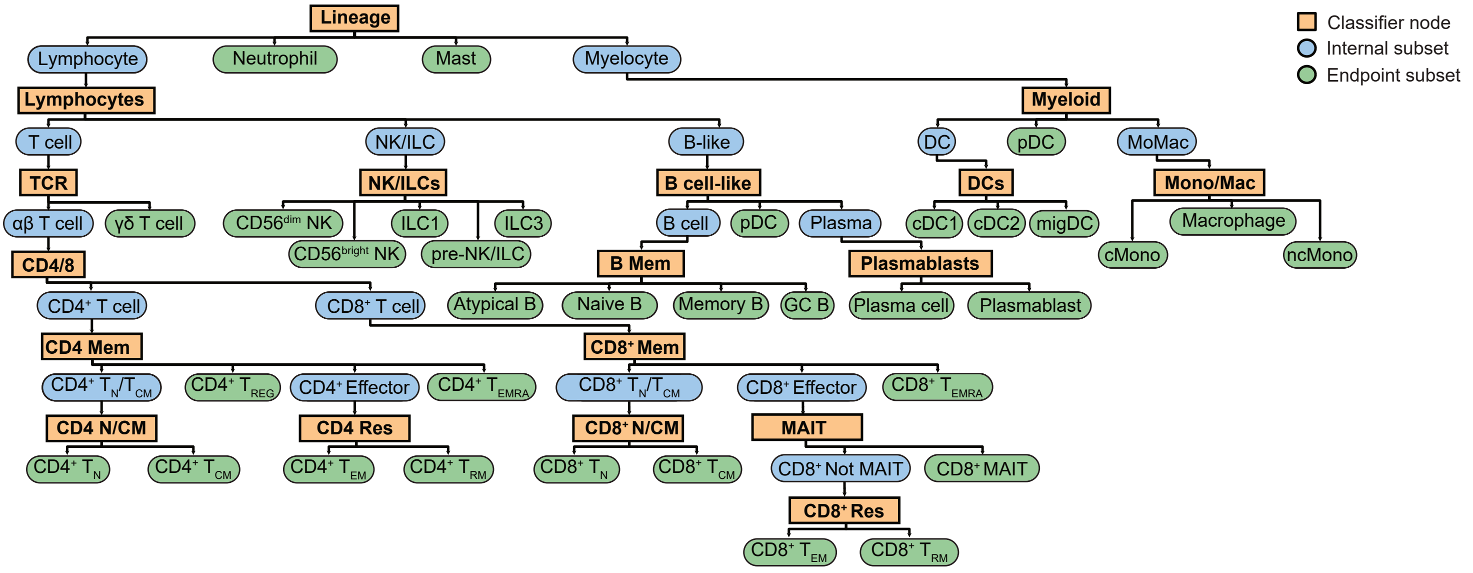

Supplementary Fig. 1: MMoCHi classifier hierarchy.

Summary of the cell types classified during our automated annotation process with Multi-Modal Classifier Hierarchy (MMoCHi)[12]. Cell subsets are organized hierarchically. MMoCHi classifiers are applied at each decision point (orange nodes), in the hierarchical order in which they appear, to reach an "end point" annotation (indicated by green nodes).

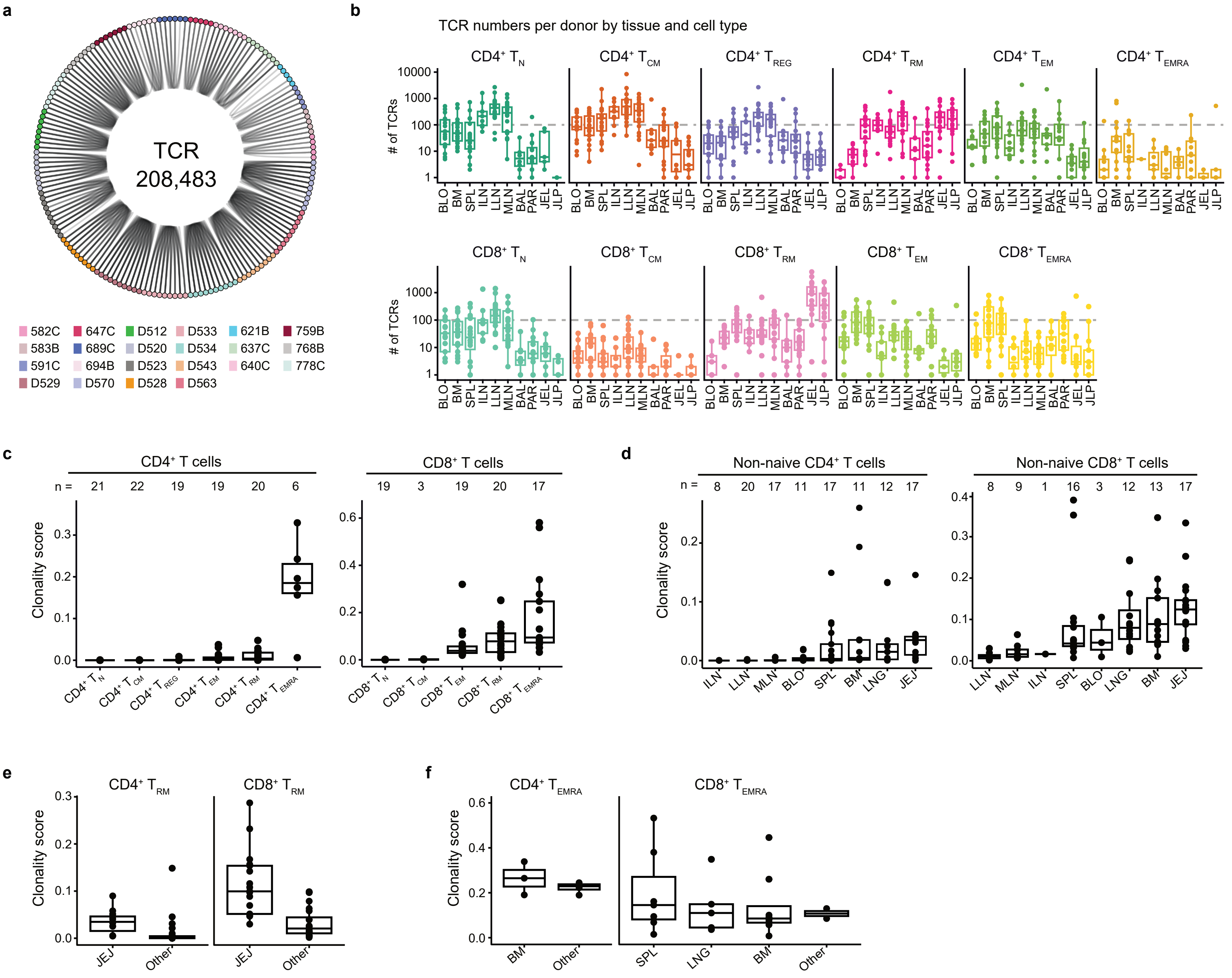

Supplementary Fig. 2: T cell clonality is cell type and tissue specific.

Summary of single-cell TCR sequences obtained from 208,483 T cells. **a)** Circle map showing clonal overlap within and across donors. Each dot represents one sample and is colored by the donor of origin. The vast majority of TCR clones are shared within a donor. **b)** Boxplots displaying the number of TCR clones identified from each MMoCHi-defined subset and tissue, with each dot representing a donor. The dotted horizontal line indicates 100 cells—the minimum cut off for inclusion in subsequent analyses. **c-d)** Comparison of clonality scores (1−Pielou's evenness index) across cell types **(c)** or tissues **(d)**. Low clonality scores correspond to high clonal diversity. Number of samples within each cell-type or tissue displayed above each plot. **e-f)** Clonality scores compared across tissues as in **(d)**, limited to either only T_{RM} cells **(e)** or only T_{EMRA} cells **(f)** to control for compositional shifts across tissues.

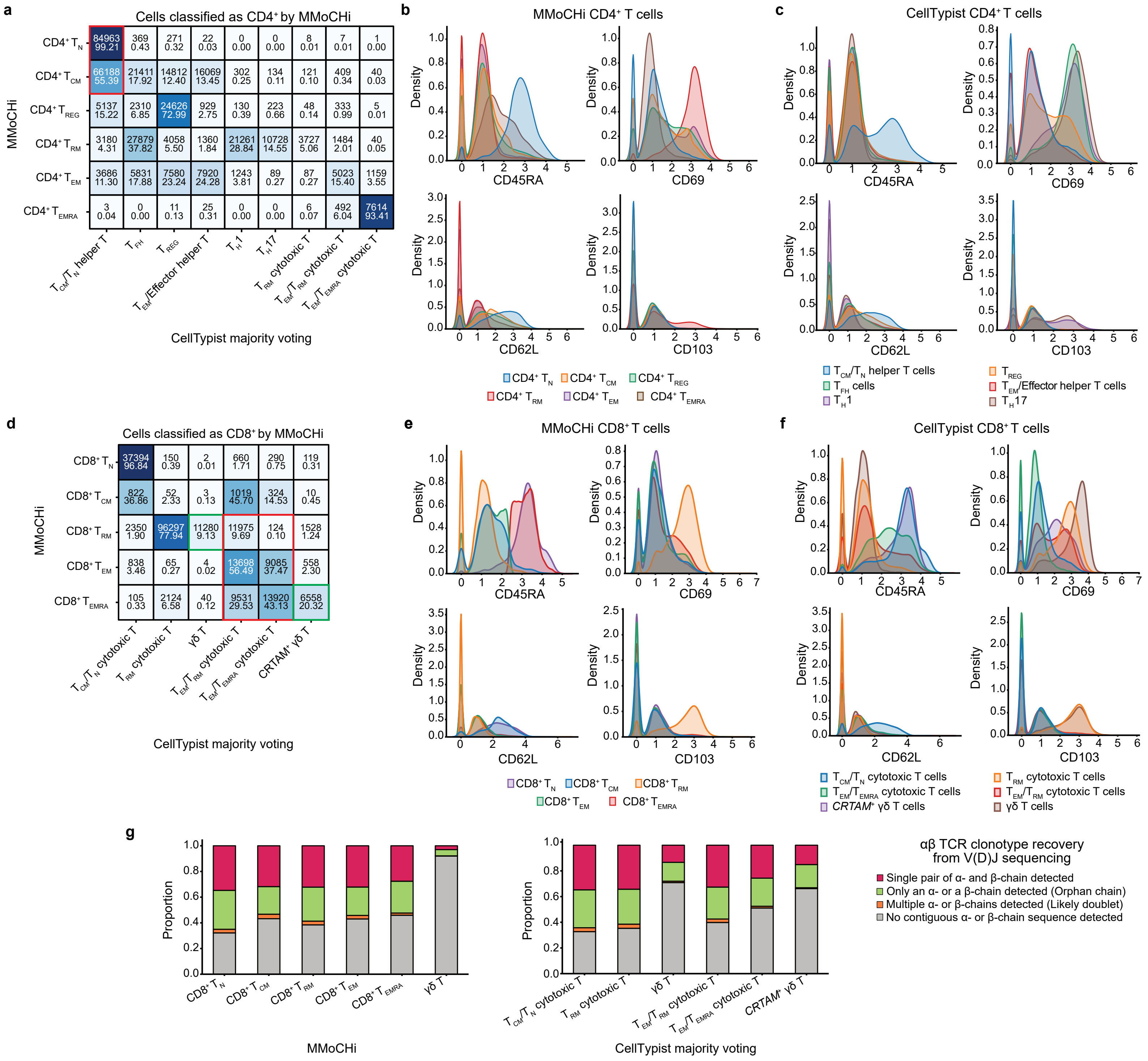

Supplementary Fig. 3: Multimodal classification improves discrimination of CD4$^+$ and CD8$^+$ T cell subsets. **a)** Heatmap displaying concordance of MMoCHi (multimodal classification) and CellTypist (classification on gene-expression alone) annotations for all cells classified as a CD4$^+$T cell by MMoCHi. The upper value shows the number of cells and the lower the percent of CellTypist annotations that are shared with MMoCHi classification. CellTypist has joint labels for some T cell subsets which can be further resolved by MMoCHi by using surface protein expression (boxed in red). Only CellTypist labels detected with greater than 1% frequency are shown, and rows sum to 100%. **b-c)** Density plots showing expression of CD45RA, CD62L, CD69 and CD103 across MMoCHi defined **(b)** or CellTypist defined **(c)** CD4$^+$ T cell subset. **d-f)** Same as **(a-c)**, but for CD8$^+$T cells. **g)** Proportion of events within each subset with an αβ TCR detected by V(D)J sequencing. A proportion of MMoCHi-labeled CD8$^+$T$_{RM}$ and CD8$^+$T$_{EMRA}$ cells were annotated as γδ T cells by CellTypist (boxed in green), but reveal high frequencies of detected αβ TCRs.

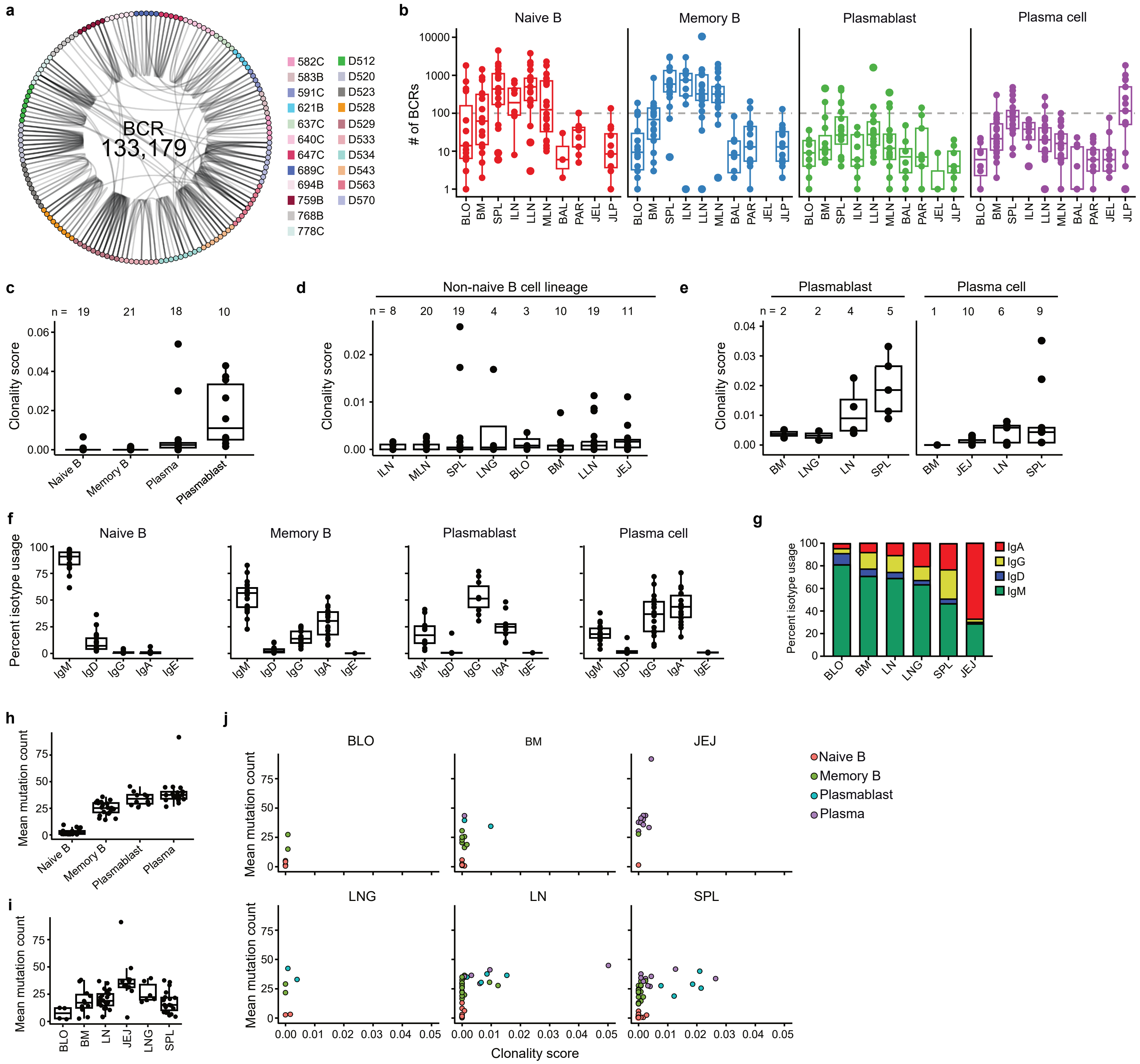

Supplementary Fig. 4: Somatic hypermutation and clonal expansion in plasma cells and plasmablasts. Summary of single-cell BCR sequences obtained from 133,179 B cells. **a)** Circle map showing clonal overlap within and across donors. Each dot represents one sample and is colored by the donor of origin. The vast majority of BCR clones are shared within a donor. **b)** Boxplots displaying the number of BCR clones identified from each MMoCHi-defined subset and tissue, with each dot representing a donor. The dotted horizontal line indicates 100 cells—the minimum cut off for inclusion in subsequent analyses. **c-d)** Comparison of clonality scores across cell types **(c)** or tissues **(d)**. Low clonality scores correspond to high clonal diversity. Number of samples within each cell-type or tissue displayed above each plot. **e)** Clonality scores compared across tissues as in **(d)**, limited to either only plasmablasts or only plasma cells to control for compositional shifts across tissues. **f-g)** Comparison of isotype usage (as determined from the BCR sequence) in each cell type **(f)** or tissue **(g)**. The IgE isotype was excluded from tissue-analysis as the frequency was less than 0.5% in each tissue. **h-i)** Mean number of somatic hypermutations detected within each cell type **(h)** or tissue **(i)**. **j)** Visualization of the relationship between clonality score and mean mutation count, score.

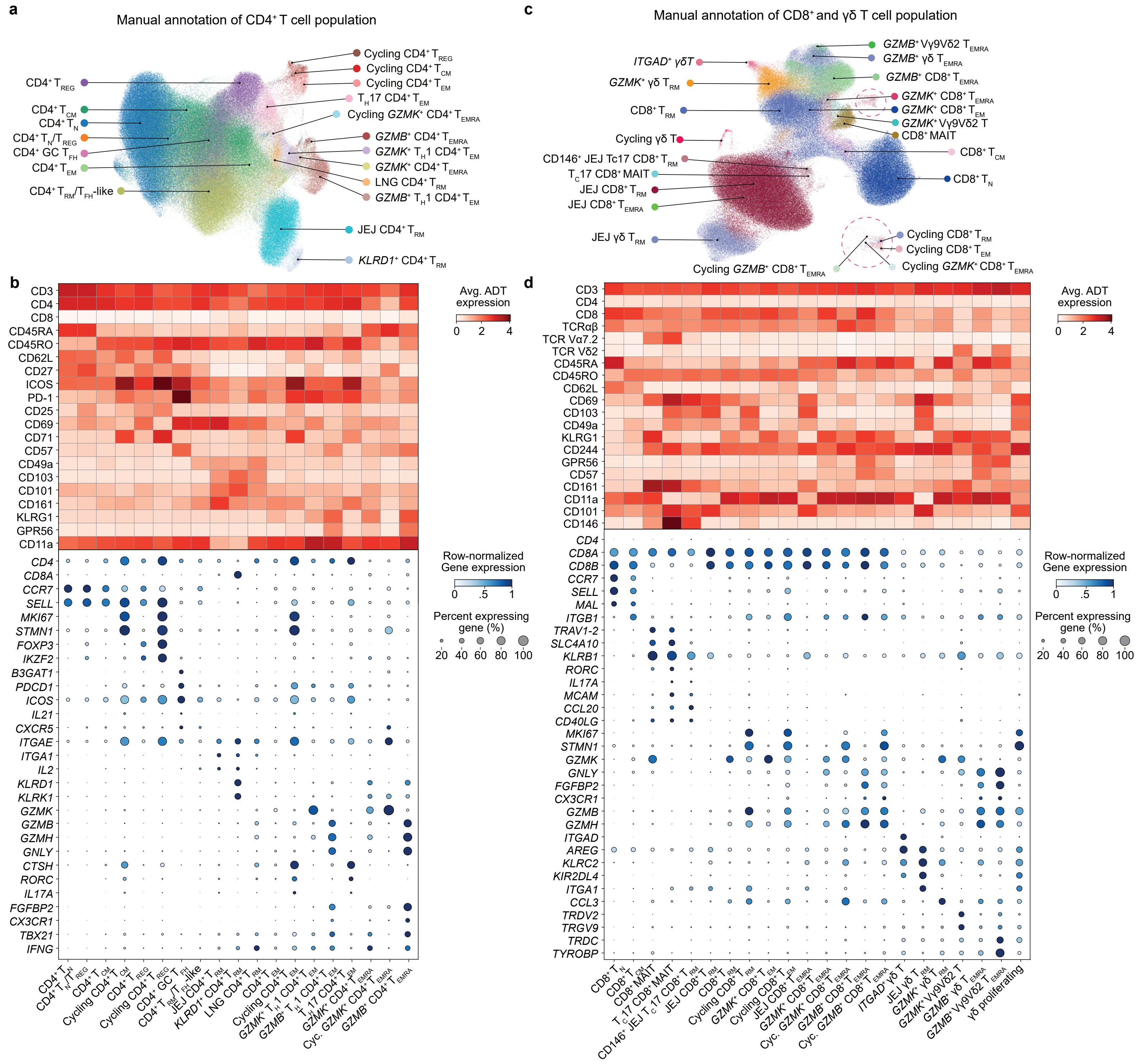

Supplementary Fig. 5: Manual annotation of T cell populations identified by the MMoCHi classifier. **a-d)** Manual annotation of unsupervised clustering was performed to identify underlying heterogeneity within MMoCHi-classified CD4^{+}T cells **(a, b)** and CD8^{+}T cells **(c, d)**. Lineage-specific UMAP embeddings are colored by annotated subset. **(b, d)** Heatmaps of immune subsets showing landmark-registered surface protein expression (*top*) and dot plots of row-normalized gene expression (log(CP10k+1); *bottom*) for each subset. Dot size corresponds to the percentage of cells within a population that has any expression of the transcript.

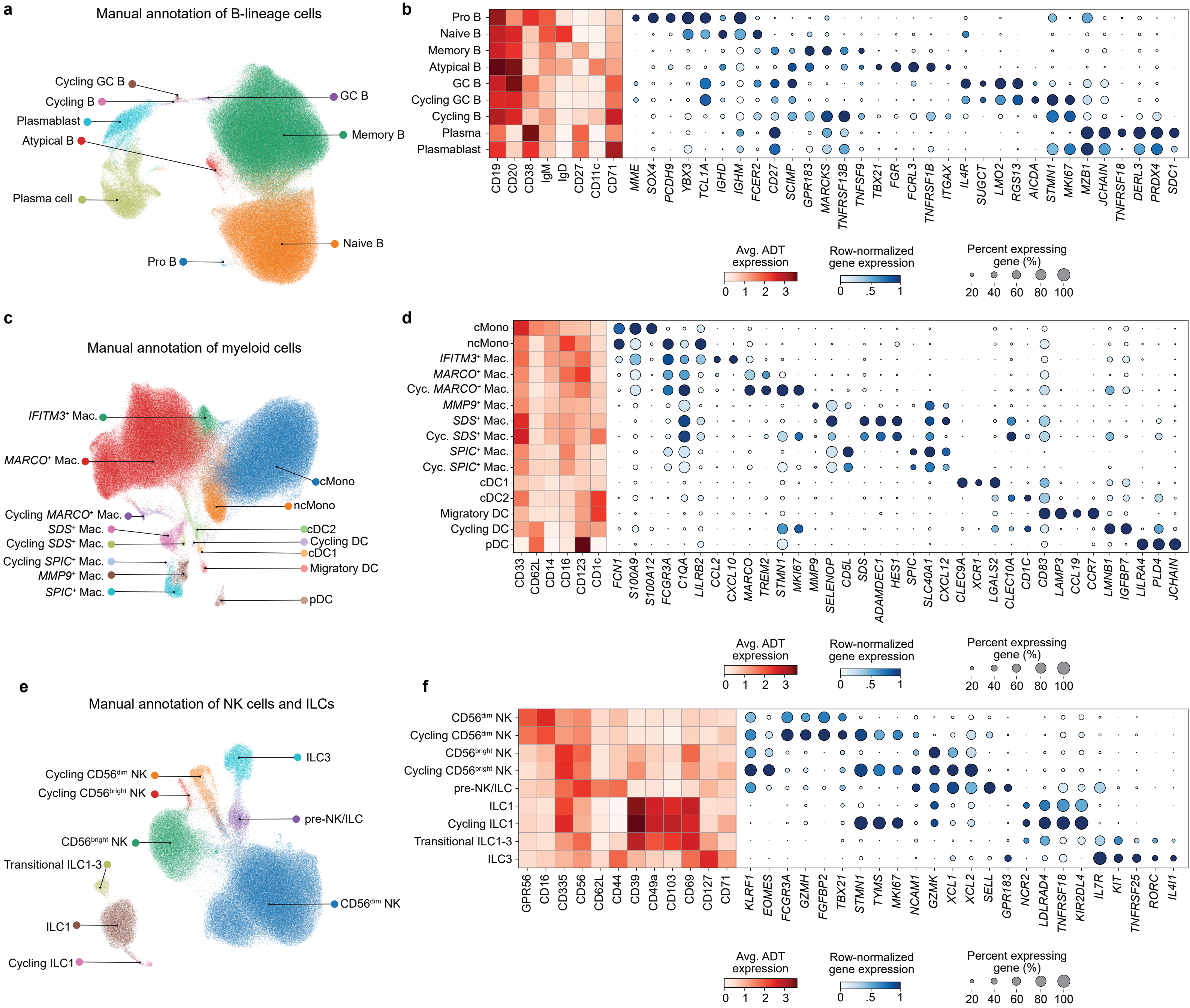

Supplementary Fig. 6: Manual annotation of immune subsets identified by the MMoCHi classifier.
a-f) Manual annotation of unsupervised clustering was performed to identify underlying heterogeneity within MMoCHi-classified B cells **(a, b)**, Myeloid cells **(c, d)**, or NK/ILCs **(e, f)**. Lineage-specific UMAP embeddings **(a, c, e)** are colored by annotated subset. Heatmaps **(b, d, f)** of immune subsets showing landmark-registered surface protein expression (*left*) and dot plots of row-normalized gene expression (log(CP10k+1); *right*) for each subset. Dot size corresponds to the percentage of cells within a population that has any expression of the transcript.

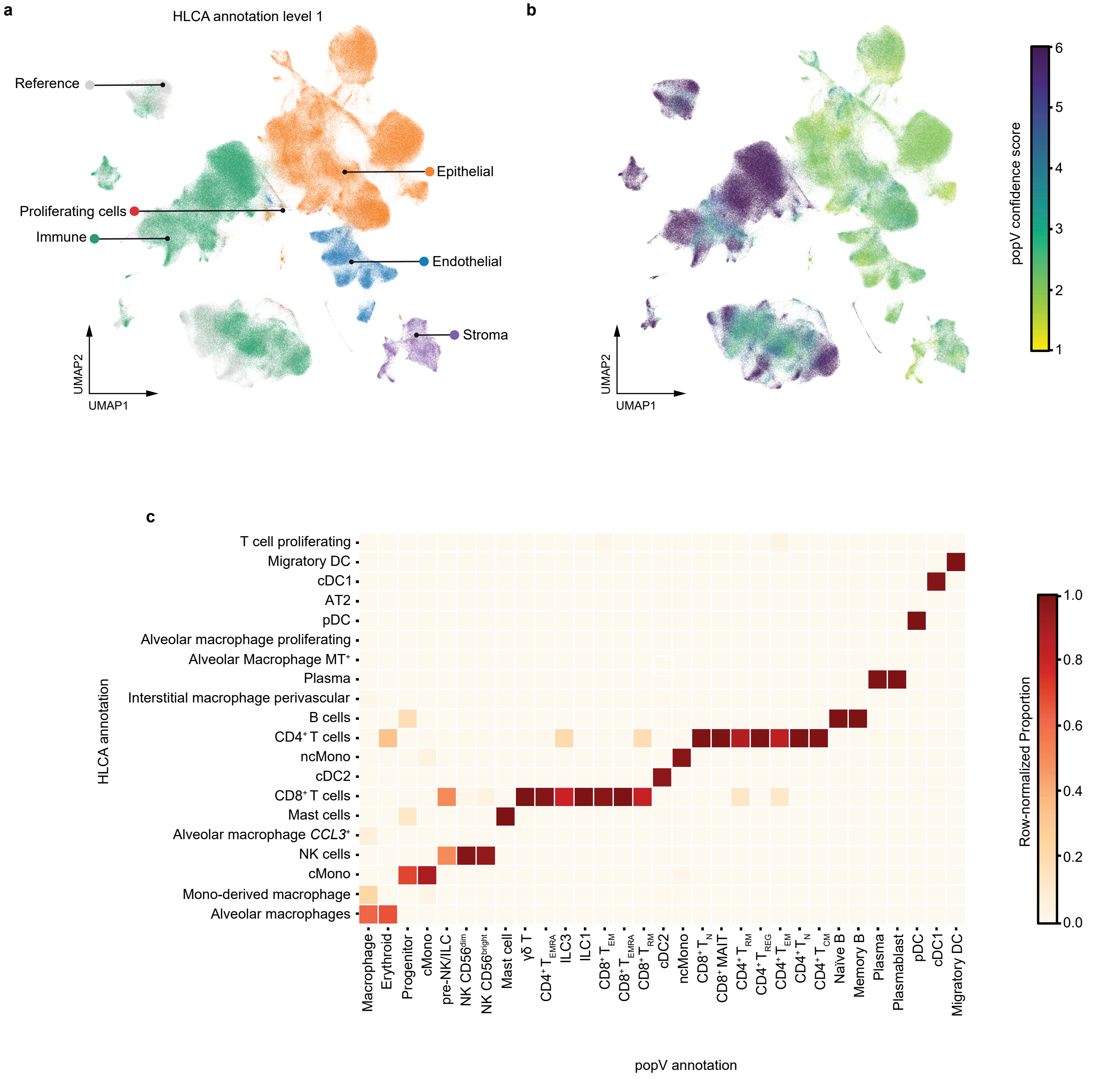

Supplementary Fig. 7: Leveraging our annotation to re-annotate the Human Lung Cell Atlas using popV. **a)** We trained popV using our data (using the MMoCHi annotations) as the reference data set and the Human Lung Cell Atlas (HLCA)[90] as the query dataset. A joint scVI embedding (calculated as part of the popV pipeline) is presented as a UMAP with the reference cells in grey and the query cells colored by their classification into major cell types (as provided by HLCA; note that these original annotations were not used in our analysis). **b)** UMAP embedding of the joint data colored by popV confidence levels (1: low confidence; 6: high). popV highlights non-immune cells as annotated at low confidence, which is expected as these cells are not present in our dataset. **c)** Heatmap comparing popV annotations (columns) to the original HLCA annotations in the study (rows) showing a high level of concordance. Specifically, our dendritic cell subset annotations (automated) overlap with the curated HLCA labels, highlighting the quality of label transfer. In other lineages, popV is able to resolve additional subtypes for B cells, NK cells, and T cells. Notably, in labelling T cells (bottom left cluster on the UMAP), popV has relatively low confidence (compared to other immune lineages) since distinguishing between some subtypes (e.g. T_{CM} and T_N) is difficult using gene expression alone (as available in the HLCA study).

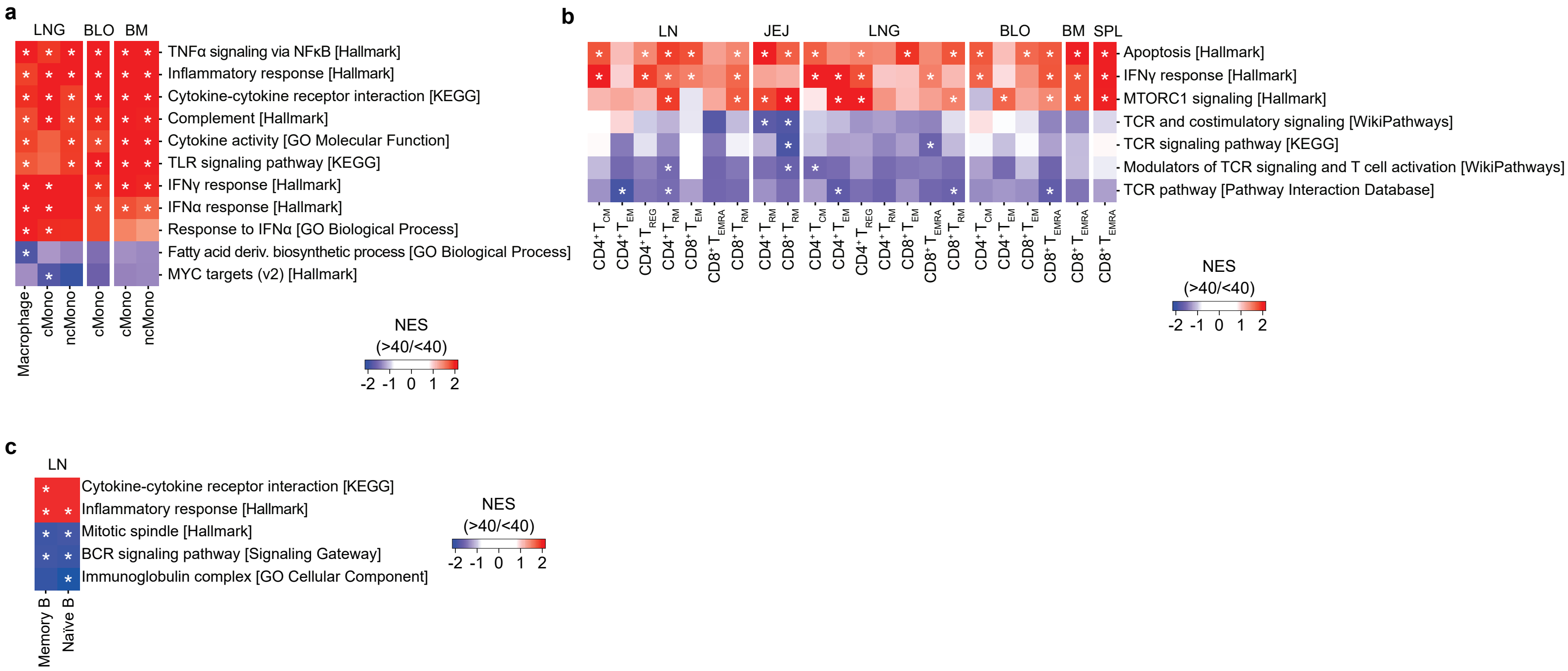

Supplementary Fig. 8: Signatures of immune aging across tissues and subsets.
GSEA was used to perform pathway analysis on pseudobulk DE across age (>40 y.o./<40 y.o.). Pathways from various sources (in brackets) shown if significant in one or more subset/tissue combinations. **a-c)** Heatmaps displaying normalized enrichment score for pathways identified in **(a)** monocytes and macrophages, **(b)** T cells, and **(c)** B cells. * denotes an adj. p-val (FDR) < 0.05.

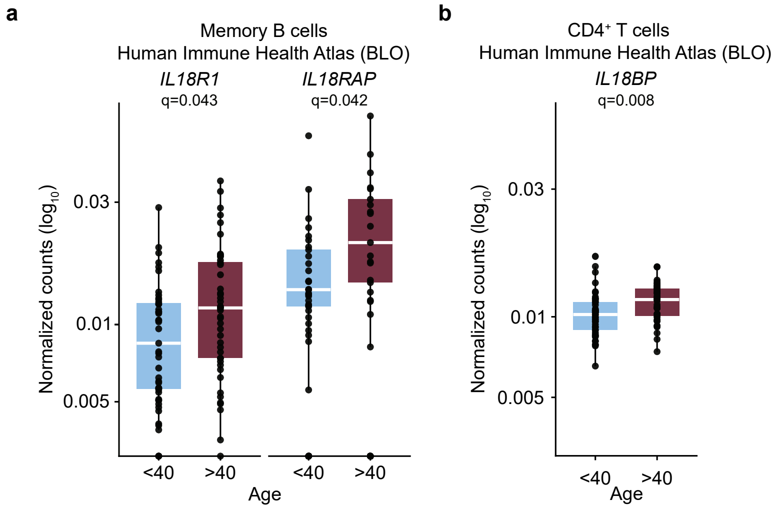

Supplementary Fig. 9: Interrogation of IL-18 Pathway genes in Human Immune Health Atlas.
a) Expression of IL-18 pathway genes with sufficient detection in memory B cells, comparing younger (<40 years) and older (>40 years) donors. **b)** *IL18BP* expression in $CD4^{+}$ T cells, comparing younger (<40 years) and older (>40 years) donors. Statistical significance by two-sided Wilcoxon rank sum followed by multiple comparisons correction (FDR).

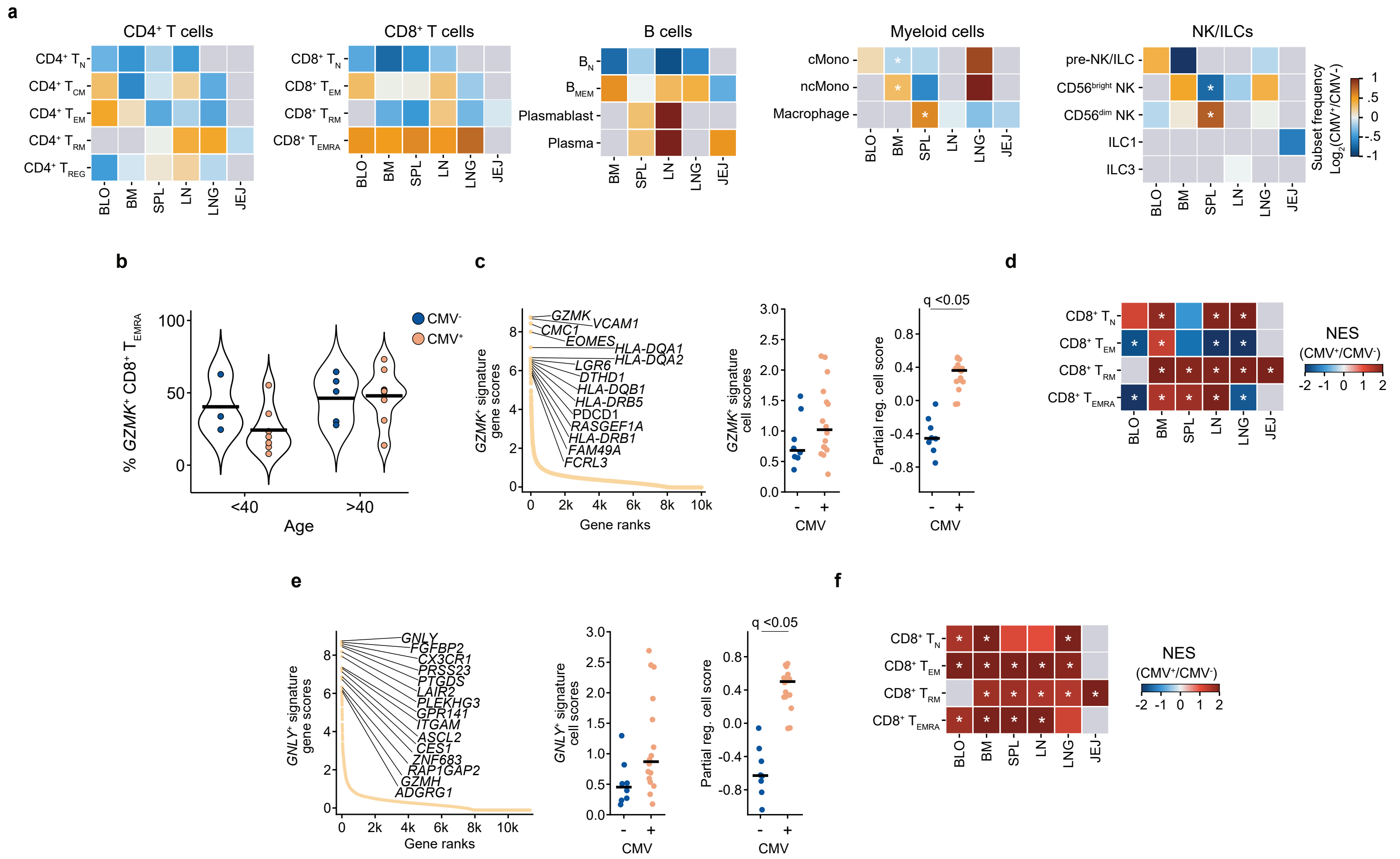

Supplementary Fig. 10: Effect of CMV serostatus on cell composition and aging signatures.
a) Heatmaps of immune subset composition changes by CMV serostatus across tissues. (LFC CMV^-/CMV^+) assessed by multivariate linear regression. **b)** Violin plots of *GZMK*$^+$CD8$^+$T$_{EMRA}$ frequencies across donors by age and CMV serostatus. **c)** Consensus scHPF factor for the *GZMK*$^+$CD8$^+$T cell signature. Dot plots show gene ranks and cell scores by CMV serostatus group, with linear mixed modeling used for the adjusted cell scores. **d)** Heatmap showing normalized enrichment scores (NES) from pre-ranked GSEA using top 200 genes from the *GZMK*$^+$ signature as gene set in CD8$^+$T cell subset-level DE across CMV serostatus. **e)** Consensus scHPF factor for the CD8$^+$T cell signature #4. Dot plots show gene ranks and cell scores by CMV serostatus group, with linear mixed modeling used for the adjusted cell scores. **f)** Heatmap showing normalized enrichment scores (NES) from pre-ranked GSEA using top 200 genes from CD8$^+$T cell signature #4 as gene set in CD8$^+$T cell subset-level DE across CMV serostatus. * denotes an adj. p-val (FDR) < 0.05.
