# Supplementary material for: Multimodal profiling reveals tissue-directed signatures of human immune cells altered with age
Source: Nat Immunol. 2025 Aug 13;26(9):1612–25. doi: 10.1038/s41590-025-02241-4 (PMC12396968; doi:10.1038/s41590-025-02241-4)
Supplement: Supplementary file 2 — Reporting Summary [file 41590_2025_2241_MOESM2_ESM.pdf]

nature portfolio

Corresponding author(s): Donna Farber, Nir Yosef, Peter Sims, Joanne Jones, Sarah Teichmann

Last updated by author(s): Jun 26, 2025

Reporting Summary

Nature Portfolio wishes to improve the reproducibility of the work that we publish. This form provides structure for consistency and transparency in reporting. For further information on Nature Portfolio policies, see our Editorial Policies and the Editorial Policy Checklist.

Statistics

For all statistical analyses, confirm that the following items are present in the figure legend, table legend, main text, or Methods section.

n/a	Confirmed	
☐	☒	The exact sample size (*n*) for each experimental group/condition, given as a discrete number and unit of measurement
☒	☐	A statement on whether measurements were taken from distinct samples or whether the same sample was measured repeatedly
☐	☒	The statistical test(s) used AND whether they are one- or two-sided<br>*Only common tests should be described solely by name; describe more complex techniques in the Methods section.*
☐	☒	A description of all covariates tested
☐	☒	A description of any assumptions or corrections, such as tests of normality and adjustment for multiple comparisons
☐	☒	A full description of the statistical parameters including central tendency (e.g. means) or other basic estimates (e.g. regression coefficient) AND variation (e.g. standard deviation) or associated estimates of uncertainty (e.g. confidence intervals)
☐	☒	For null hypothesis testing, the test statistic (e.g. *F*, *t*, *r*) with confidence intervals, effect sizes, degrees of freedom and *P* value noted<br>*Give P values as exact values whenever suitable.*
☒	☐	For Bayesian analysis, information on the choice of priors and Markov chain Monte Carlo settings
☐	☒	For hierarchical and complex designs, identification of the appropriate level for tests and full reporting of outcomes
☐	☒	Estimates of effect sizes (e.g. Cohen's *d*, Pearson's *r*), indicating how they were calculated

Our web collection on statistics for biologists contains articles on many of the points above.

Software and code

Policy information about availability of computer code

Data collection	Cell Ranger v6.0.0, 10x Genomics
Data analysis	Code for data processing and downstream analysis is available at the Github Repository https://github.com/YosefLab/CZI-Immuneaging.<br><br>Scipy v1.6.3<br>decontX v1.7.3<br>scikit-learn v0.24.2<br>CellTypist v0.9<br>scvi-tools v0.14.5<br>HashSolo v1.7.2<br>Scrublet v1.7.2<br>Scanpy v1.7.2<br>MMocHi v0.2.1<br>Dreamlet v1.4.1<br>scHPF v0.1<br>mrVI v0.3<br>Decoupler v1.3<br>blastn v2.13.0<br>IgBLAST v1.19.0<br>TIgGER v1.0.1<br>Dandelion v0.3.1

seaborn v0.13.2
statsmodels v0.14.1
gseapy v1.1.3
scCODA v0.1.9
popV v0.5.1
AnnData v0.9.2

For manuscripts utilizing custom algorithms or software that are central to the research but not yet described in published literature, software must be made available to editors and reviewers. We strongly encourage code deposition in a community repository (e.g. GitHub). See the Nature Portfolio guidelines for submitting code & software for further information.

Data

Policy information about availability of data

All manuscripts must include a data availability statement. This statement should provide the following information, where applicable:

- Accession codes, unique identifiers, or web links for publicly available datasets
- A description of any restrictions on data availability
- For clinical datasets or third party data, please ensure that the statement adheres to our policy

The global, T, NK/ILC, B and Myeloid cells h5ad datasets are available at Lattice : Human Cell Atlas https://cellxgene.cziscience.com/collections/cc431242-35ea-41e1-a100-41e0dec2665b. Raw sequencing (Fastq) files are available in Sequence read archive (SRA) under accession SRP559768 and BioProject accession PRJNA1215450. Data are also available in gene expression omnibus under accession number GSE299043.

Research involving human participants, their data, or biological material

Policy information about studies with human participants or human data. See also policy information about sex, gender (identity/presentation), and sexual orientation and race, ethnicity and racism.

Reporting on sex and gender	Sex of human organ donors was provided by organ procurement organization or biorepository and indicated in the manuscript (14 males, 10 females). When appropriate, sex was included as a covariate in the analysis. Gender was not provided or considered in study design.
Reporting on race, ethnicity, or other socially relevant groupings	Race, ethnicity, or other socially relevant groupings were not used in the manuscript.
Population characteristics	Deceased human organ donors from 20-75 years of age, both male and female, and CMV+ and CMV- serology. All donors were free of cancer, chronic disease, seronegative for hepatitis B, C and HIV and did not show evidence for active infection based on blood, urine, respiratory and radiological surveillance testing. All covariates are noted in the manuscript.
Recruitment	Donors were identified through collaborations with the LiveOnNY organ procurement organization or through the Cambridge Biorepository for Translational Medicine.
Ethics oversight	Human tissues were obtained from deceased organ donors and therefore not subject to IRB assurances for human subjects protections. Donors from New York, USA were obtained through a materials transfer agreement with LiveOnNY and donors from Cambridge, UK were obtained from the Cambridge Biorepository for Translational Medicine (REC 15/EE/0152).

Note that full information on the approval of the study protocol must also be provided in the manuscript.

Field-specific reporting

Please select the one below that is the best fit for your research. If you are not sure, read the appropriate sections before making your selection.

☒ Life sciences ☐ Behavioural & social sciences ☐ Ecological, evolutionary & environmental sciences

For a reference copy of the document with all sections, see nature.com/documents/nr-reporting-summary-flat.pdf

Life sciences study design

All studies must disclose on these points even when the disclosure is negative.

Sample size	Data from 24 human organ donors were acquired from all tissues that were available for research purposes. Sample size was determined based on tissue availability and representation of donors > or < 40 years of age.
Data exclusions	scRNA-seq from the skin, liver and colon were acquired on select donors and removed from downstream analysis due to low cell or donor number. Non-immune cells were also removed for analysis as the focus of the study was on the immune system.

Replication	Where possible, data from individual donors were treated as independent observations to ensure robustness and reproducibility. This study involves profiling of immune cells from up to 14 sites of 24 individual donors. Replication was based on site and donor.
Randomization	All samples acquired from deceased human organ donors were analyzed, no randomization was required.
Blinding	This study involves immune cell profiling from multiple tissue and donors isolated directly from live human samples. It is an exploratory study and did not require blinding for outcomes because there were not experiments set up to obtain outcomes.

Reporting for specific materials, systems and methods

We require information from authors about some types of materials, experimental systems and methods used in many studies. Here, indicate whether each material, system or method listed is relevant to your study. If you are not sure if a list item applies to your research, read the appropriate section before selecting a response.

Materials & experimental systems

n/a	Involved in the study	
☐	☒	Antibodies
☒	☐	Eukaryotic cell lines
☒	☐	Palaeontology and archaeology
☒	☐	Animals and other organisms
☒	☐	Clinical data
☒	☐	Dual use research of concern
☒	☐	Plants

Methods

n/a	Involved in the study	
☒	☐	ChIP-seq
☒	☐	Flow cytometry
☒	☐	MRI-based neuroimaging

Antibodies

Antibodies used

Biotin anti-human CD235ab Antibody (100μg) (Biolegend, Cat. No.: 306618)
Biotin anti-human CD66b Antibody (100μg) (Biolegend, Cat. No.: 305120)
Biotin anti-human CD326 (EpCAM) Antibody (100μg) (Biolegend, Cat. No.: 324216)
TotalSeq™-A0251 anti-human Hashtag 1 Antibody (BioLegend, Cat. No.: 394601)
TotalSeq™-A0252 anti-human Hashtag 2 Antibody (BioLegend, Cat. No.: 394603)
TotalSeq™-A0253 anti-human Hashtag 3 Antibody (BioLegend, Cat. No.: 394605)
TotalSeq™-A0254 anti-human Hashtag 4 Antibody (BioLegend, Cat. No.: 394607)
TotalSeq™-A0255 anti-human Hashtag 5 Antibody (BioLegend, Cat. No.: 394609)
TotalSeq™-A0256 anti-human Hashtag 6 Antibody (BioLegend, Cat. No.: 394611)
TotalSeq™-A0257 anti-human Hashtag 7 Antibody (BioLegend, Cat. No.: 394613)
TotalSeq™-A0258 anti-human Hashtag 8 Antibody (BioLegend, Cat. No.: 394615)
TotalSeq™-C0251 anti-human Hashtag 1 Antibody (BioLegend, Cat. No.: 394661)
TotalSeq™-C0252 anti-human Hashtag 2 Antibody (BioLegend, Cat. No.: 394663)
TotalSeq™-C0253 anti-human Hashtag 3 Antibody (BioLegend, Cat. No.: 394665)
TotalSeq™-C0254 anti-human Hashtag 4 Antibody (BioLegend, Cat. No.: 394667)
TotalSeq™-C0255 anti-human Hashtag 5 Antibody (BioLegend, Cat. No.: 394669)
TotalSeq™-C0256 anti-human Hashtag 6 Antibody (BioLegend, Cat. No.: 394671)
TotalSeq™-C0257 anti-human Hashtag 7 Antibody (BioLegend, Cat. No.: 394673)
TotalSeq™-C0258 anti-human Hashtag 8 Antibody (BioLegend, Cat. No.: 394675)
TotalSeq™-C0259 anti-human Hashtag 9 Antibody (BioLegend, Cat. No.: 394677)
TotalSeq™-C0260 anti-human Hashtag 10 Antibody (BioLegend, Cat. No.: 394679)
TotalSeq™-A Custom Human Panel (BioLegend, Cat. No.: 99786)
TotalSeq™-C Human Universal Cocktail, V1.0 (BioLegend, Cat. No.: 399905)

Validation

Antibody cocktails consist of well-validated lyophilized antibodies for cell surface markers at optimized concentrations for single cell sequencing analyses provided by BioLegend and used as directed at one vial per test. All hashtags were used at a 1:100 dilution, all biotinylated antibodies were used at 1:20 dilutions. The manufacturer has validated antibody cocktails on human PBMCs and we have confirmed their effiicacy on human organ donor tissues in pilot experiments and previous studies. Each lot of biotinylated and hashtag antibodies were quality control tested by immunofluorescent staining with flow cytometric analysis and the for the hashtags the oligomer sequence is confirmed by sequencing.

Plants

Seed stocks	*Report on the source of all seed stocks or other plant material used. If applicable, state the seed stock centre and catalogue number. If plant specimens were collected from the field, describe the collection location, date and sampling procedures.*
Novel plant genotypes	*Describe the methods by which all novel plant genotypes were produced. This includes those generated by transgenic approaches, gene editing, chemical/radiation-based mutagenesis and hybridization. For transgenic lines, describe the transformation method, the number of independent lines analyzed and the generation upon which experiments were performed. For gene-edited lines, describe the editor used, the endogenous sequence targeted for editing, the targeting guide RNA sequence (if applicable) and how the editor was applied.*
Authentication	*Describe any authentication procedures for each seed stock used or novel genotype generated. Describe any experiments used to assess the effect of a mutation and, where applicable, how potential secondary effects (e.g. second site T-DNA insertions, mosiacism, off-target gene editing) were examined.*
